# Exploring the challenge of health research priority setting in partnership: reflections on the methodology used by the James Lind Alliance Pressure Ulcer Priority Setting Partnership

**DOI:** 10.1186/s40900-016-0026-y

**Published:** 2016-04-02

**Authors:** Mary Madden, Richard Morley

**Affiliations:** 1 School of Healthcare, Faculty of Medicine and Health, University of Leeds, 2.19, Baines Wing, Leeds, LS2 9JT UK; 2 Consumer Network Coordinator, Cochrane, St Albans House, 57-59 Haymarket, London, SW1Y 4QX UK

**Keywords:** Research priority setting, Evidence-informed healthcare, Wound care, PPI methodology, The James Lind Alliance, Value in research

## Abstract

**Plain English Summary:**

The James Lind Alliance (JLA) brings patients, carers and clinicians together in Priority Setting Partnerships (PSPs) to identify and prioritise shared uncertainties about the effects of treatment. The JLA emerged from the evidence-informed healthcare movement to address a concern that the research being carried out on treatment effectiveness is not that of most importance to patients and health professionals. In the JLA PSPs, ‘hard’ evidence-informed ideals meet ‘soft’ participatory practices. This article explores the challenges of putting co-production methods into practice by reflecting on the methods used by the JLA Pressure Ulcer PSP (JLAPUP). The JLA principles are transparency, inclusivity and avoiding waste in research. This means paying the same close critical attention to how PSPs are designed and run, as is desired in the health research which the JLA seeks to influence. JLAPUP showed that it was possible to work in partnership in a field where patients are often elderly, immobile, unrepresented and particularly unwell, many of whom are living with more than one long term condition. However, for those unfamiliar with it, ‘uncertainty’ was a difficult term to get to grips with. Also, it was harder for some people than others to take part and to have their voices heard and understood. In keeping with other PSPs, JLAPUP found that the nature and quality of research into pressure ulcer prevention and treatment did not reflect the priorities of those who took part.

**Abstract:**

ᅟ

**Background:**

Studies identifying a mismatch between the priorities of academics and clinicians and those of people with direct experience of a health condition pose a challenge to the assumption that professional researchers can represent the interests of patients and the public in setting priorities for health research. The James Lind Alliance (JLA) brings patients, carers and clinicians together in Priority Setting Partnerships (PSPs) to identify and prioritise shared uncertainties about the effects of treatment. There is no formal evaluation yet to examine the different approaches used by individual PSPs and the impact these methods have on the quality of the partnership and subsequent outputs. There is no gold standard method for health research topic identification and priority setting and reporting on public involvement in this area is predominantly descriptive rather than evaluative.

**Methods and Findings:**

The JLA Pressure Ulcer PSP (JLAPUP) was developed and worked between 2009 and 2013 to identify and prioritise the top 10 ‘uncertainties’**,** or ‘unanswered questions’, about the effects of pressure ulcer interventions. JLAPUP identified a mismatch between the nature and quality of RCTs in pressure ulcer prevention and treatment and the kind of research evidence desired by patients or service users, carers and health professionals. Results and methods have been reported fully elsewhere. The consultative and deliberative methods used to establish health research priorities in PSPs are fundamentally interpretive. PSPs are therefore an arena in which ‘hard’ evidence-informed ideals meet ‘soft’ participatory practices. This article provides an account of the challenges faced in one particular PSP. We explain the rationale for the approaches taken, difficulties faced and the limitations at each stage, because these aspects are particularly under-reported. The JLAPUP case is used to identify possible areas for evaluation and reporting across PSPs.

**Conclusion:**

Engaging people with very different health and life experiences in the complexities of health science based discussions of uncertainty is challenging. This is particularly the case when engaging groups routinely excluded from participating in health research, for example, older people with multiple comorbidities. The JLA principles of transparency, inclusivity and avoiding waste in research require paying close critical attention to PSP methodology, including full evaluation and reporting of PSP processes and outcomes. Assessing the impact of PSPs is contingent on the decision making processes of commissioners and funders.

## Background

Studies identifying a mismatch between the priorities of academics and clinicians and those of people with direct experience of a health condition pose a challenge to the assumption that professional researchers can represent the interests of patients and the public in setting priorities for health research [1, 2]. The James Lind Alliance (JLA) brings patients, carers and health professionals together in Priority Setting Partnerships (PSPs) to identify and prioritise shared uncertainties about the effects of treatment. In PSPs, ‘hard’ evidence-informed ideals inevitably meet ‘soft’ participatory practices. The aim of this article is to improve understanding of how to make health research priority setting partnerships work in practice by making transparent the challenges facing a particular JLA PSP and by discussing the strengths and weaknesses of some of the strategies used to meet those challenges.

Methods and results of the JLA Pressure Ulcer PSP (JLAPUP) have been reported fully elsewhere [3, 4]. Here, in keeping with the JLA principles of transparency and inclusivity and the evidence-informed healthcare movement principle of avoiding waste in research [5], we examine in more detail some of the challenges and limitations of the JLAPUP complex co-production process. We also explain the rationale and context for the development of the JLA and JLAPUP. The origin of the JLA in the evidence-informed healthcare movement (rather than for example patient based movements) is important in understanding its focus on ‘uncertainty’ and the randomised controlled trial (RCT). The JLAPUP case is used to identify possible areas for further evaluation and reporting across PSPs. Key issues raised for further investigation are:

- the extent to which people understand the process in which they are participating, including the concept of uncertainty as the starting point for research
- inclusions and exclusions from and within the partnership, especially its decision- making fora (Steering Groups and the final meeting) and how to engage frail elders, including care home residents, in the process
- ethical considerations, including the necessity and worth of negotiating the UK National Health Service (NHS) ethics framework
- effective survey design for consultation and prioritisation
- interpreting open-ended submissions without ‘reading into’ them
- whether final priorities are also ‘researchable questions’ and what to do with submissions not suitable for RCTs
- resources required to adequately check that there is no evidence to answer submitted questions and to update materials for the UK Database of Uncertainties about the Effects of Treatments (UK DUETs)
- the role and responsibility of a PSP in fielding: individual requests for advice about a health condition; offers of resources and involvement from industry (given increasing private involvement in public health and social care provision); and general requests to act as a mouthpiece for a perhaps otherwise poorly represented health condition
- how to promote uncertainties and assess impact when the funding runs out
- lifespans and full costings of PSPs

### The JLA and the role of the public in UK health research

The JLA was established in 2004 to encourage patients, carers and clinicians to work together to identify and prioritise shared healthcare uncertainties, arguing that research into clinical practice and national health services should identify and address the questions and uncertainties that are of most practical importance to patients, their carers and clinicians^1^ [6, 7]. The JLA argues that medical research agendas have paid insufficient attention to the treatment questions and outcomes that matter most to those directly affected, and that those affected should hold the research community to account [8]. The JLA emerged from the wider James Lind Initiative (JLI) established in 2003 in response to a UK Medical Research Council (MRC) call for the creation of, “a communications and discussion forum on Randomised Controlled Trials (RCTs), involving patients, practitioners, researchers, and others” [9]. In 2013, the JLA was designated a partner organization of the National Institute for Health Research (NIHR) to be managed by the NIHR Evaluation, Trials and Studies Coordinating Centre (NETSCC). The JLA is also working outside the UK, for example JLA advisors have supported a PSP on dialysis in Canada [10].

The JLA emphasises working in partnership and has its origins in the evidence-informed healthcare movement rather than formal NHS and NIHR patient and public involvement (PPI) policy or patient/service user movements. PPI in UK health research is defined as research carried out with or being carried out by members of the public (or service users), rather than research on patients and members of the public in the role of subjects or participants [11]. It is a UK policy imperative and a prerequisite for many health research funders despite concerns about poor reporting, minimal theoretical or conceptual underpinning, lack of measurement of outcomes, poor evaluation and lack of attention to potential harms or adverse effects [12]. There is little empirical evidence on the extent, processes and impact of user involvement in research, and some conflict amongst practitioners about the normative, substantive and process-related principles underpinning PPI [13]. Staniszewska et al. are attempting to develop a checklist to enhance the quality of reporting and to enable more effective evaluation [14]. Thompson et al. see the ongoing emphasis on documenting the measurable impacts and justifications for PPI as suggestive of a crisis of legitimacy [15]. The extent to which UK policy support for PPI in health research extends to influencing clinical research agendas remains unclear [16].

The empirical evidence base underpinning the worth and impact of lay involvement in research is in itself poor and requires strengthening, nevertheless there are at least three arguments for involving patients, service users and carers in deciding on research priorities, in addition to the NHS policy imperative for PPI [17–19]. A theoretical case for citizen participation incorporates ethical arguments based on notions of individual rights, civic responsibility, social justice and political accountability. A pragmatic argument suggests that involvement by stakeholders enhances the legitimacy of, and thereby increases support for, research. There is also an argument that experiential knowledge from those directly affected improves the quality and relevance of research, for example, identifying appropriate research questions, outcomes and measures and improving the clarity of communications, including the writing of consent forms and the dissemination of ideas. Peter Beresford, and Shaping our Lives, advocate direct involvement to reduce the gap between user experience and researcher interpretation [20].

There are many different approaches to health research prioritisation and no clear agreement on what might constitute best practice [21]. Nasser et al. have provided pointers to prevent wheel re-invention and to build more robust processes [22]. A recent cluster RCT to test the impact of involving patients in setting healthcare improvement priorities for chronic care at community level in Canada found that patient involvement fostered mutual influence between patients and professionals and increased agreement on common priorities. It also increased the costs of the prioritization process and the time required to reach consensus on common priorities [23].

In a bid to focus the research agenda on outcomes for patients and avoid the waste in health research that results from ignoring the needs of users of research evidence [24], each JLA PSP brings together disparate individuals, and organisations representing patients, carers and clinicians, in a task-focused collaborative partnership. A key feature is that partners work together in order to identify and agree treatment uncertainties. The inclusion of patients working with health professionals as partners arguably makes it harder for researchers and commissioners to ignore findings coming from one of these groups alone. PSPs use consultative and deliberative methods to identify uncertainties, interpret these as potential research questions, check the evidence base to see if these questions are already answered, deliberate and agree research priorities and promote a top ten to potential researchers and funders.

The JLA process invites partners to identify the methods they deem appropriate for soliciting, from members of the partnership, uncertainties which are of practical clinical importance about the treatment and management of a health problem. Prioritisation methods identified as potentially useful include: adapted Delphi techniques; expert panels or nominal group techniques; consensus development conference; electronic nominal group and online voting; interactive research agenda setting and focus groups [25]. A key common feature is that the final stage of prioritisation takes place at a face-to-face meeting using group discussions and plenary sessions. The key sites for the deliberative part of the process are this final priority setting meeting and the Steering Group. Confirmed uncertainties are published on the UK Database of Uncertainties about the Effects of Treatments (UK DUETs) [26]. UK DUETs was also initiated by JLI [27] and from 2010 until January 2016 was supplied and funded by the National Institute for Health and Care Excellence [28].

### Origins and challenges of developing JLAPUP

JLAPUP was initiated as part of a NIHR study, Wounds Research for Patient Benefit (WRPB) funded under its Programme Grants for Applied Research funding scheme (RP-PG-0407-10428) [3]. The objectives of this part of the programme were to identify patient/service user, carer and health professional research priorities and to investigate methodology for PPI in research priority setting in wound care. The patient and service user voice is notably absent from wounds research and in particular from conversations about outcomes [29]. WRPB approached the JLA to explore the possibility of establishing a PSP, choosing pressure ulcers because this would confront the hardest challenges of PPI work in wound care and because they are the most frequent type of complex, chronic wound [30]. A methodological review of wounds RCTs carried out by the WRPB team and colleagues found that despite the prevalence of pressure ulcers, only 19 % of recent chronic wounds trials investigated pressure ulcers and many of those trials were of poor quality [31].

A preliminary meeting between WRPB and JLA [32] explored assumptions and expectations about the types of professionals and public to be involved in developing a JLA PSP in pressure ulceration, the knowledge partners might bring to bear and methods used by other PSPs. This discussion noted the following particular challenges:

- A significant proportion of people with pressure ulcers find it difficult to participate in activities outside their homes due to immobility, frailty and co-morbidities. There is a history of routinely excluding much of the pressure ulcer constituency, older adults with multiple comorbidities, from medical research [33].
- There were (at that time) no pressure ulcer or wounds specific existing patient/service user groups.
- Online working may only be suitable for small numbers of patients and service users.
- In addition to self-care strategies from people at risk of pressure ulcers themselves supported by their families, most pressure ulcers are managed by nurses with referral to specialist services, including tissue viability and surgery. Those who are, for example, unable to reposition themselves rely on a range of health and social care staff for pressure ulcer prevention interventions. The professional community involved therefore spans NHS and Social Care providers including private health and social care.
- There is a lot of industry (medical device company) related activity including research activity in wound care. The JLA does not engage with industry in its processes because commercial interests may not align with scientific or public interests. This may challenge professionals used to working with industry as a major sponsor of health care research and education.
- There is not a culture of doing RCTs to assess effectiveness of treatments in wound care because most interventions are devices and the burden of evidence required is much less than for pharmaceuticals [34].
- Many professionals who work in this area may not be very research aware.

These factors indicated that in order to develop an accurate understanding of the unanswered questions important to pressure ulcer patients, service users, carers and health professionals, it might be necessary to try more than one method of soliciting uncertainties about the effects of pressure ulcer treatments and prevention interventions, and to vary methods between groups.

The JLAPUP protocol was developed in close collaboration with the JLA and informed by discussions about the emergent JLA partnership approach to health research priority setting (the JLA Guidebook was not yet available) and by a search for other approaches, in particular the Dialogue Model [35]. This latter model derives from work in programme evaluation in the Netherlands translated for use in health services research. Both the Dialogue Model and JLA approach use consultative and deliberative methods. A deliberative method is not solely a means of garnering opinion but also a means of considering different points of view and coming to reasoned decisions without coercion, manipulation or deception. There are different ways of going about this, but common to all is a component where, “participants are provided with information about the issue being considered, encouraged to discuss and challenge the information and consider each other’s views before making a final decision or recommendation for action” ([18]: 242). This process of deliberation can take place at an individual level or in meetings with others. A genuine dialogue implies that participants learn in the process and may change their opinion through listening to each other.

**Fig. 1:**
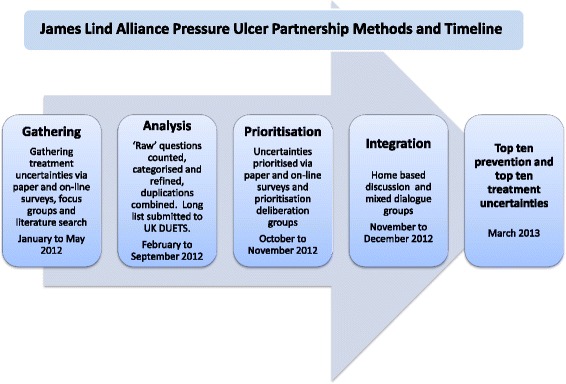
JLAPUP process flowchart

Given the absence of service user voices at the table in this sector, JLAPUP sought to maximise the opportunity for dialogue and deliberation in the priority setting process. Drawing on the available precedents, the JLAPUP protocol outlined a six phase process comprising exploration, initiation, consultation, collation, prioritisation, integration/deliberation, reporting and evaluation. The project activity timeline is detailed in Fig. 1. Some of the key questions and challenges raised by each aspect of the process are discussed below.

### Initiation: Defining and constituting the ‘pressure ulcer public’

The question of who should take part is a crucial starting point in establishing a PSP, involving consideration of how populations are being framed in research and how the PSP itself is actively forming ‘a public’. Active networking and outreach strategies are standard, but resource intensive, means of trying to engage seldom heard or (at the other extreme) overcommitted groups. It is a feature of the pressure ulcer landscape that there are few third-sector or patient led organisations with pressure ulcers as a primary concern with whom to engage. Informed judgements about inclusions and exclusions from the partnership were therefore difficult to make. This and resource constraints meant recruitment to the partnership was necessarily remote and passive, relying on interested parties to see and respond to an article or direct mailing without much follow up. A press release and invitation to an introductory workshop was circulated widely within wound care and broader health and social care networks. Social media, introduced later into the work of the PSP, offered an innovative communication tool and, if introduced earlier into the process, may have helped identify a broader range of organisations and individuals with whom to engage.

A well-attended introductory meeting was organized and hosted by WRPB, and chaired by the then JLA Co-Chair Sally Crowe. Attendees included people with direct personal experience of pressure ulcers and/or caring for others with pressure ulcers, health and PPI professionals (some of whom were also active researchers) and members of third sector organisations including Action on Elder Abuse, the Multiple Sclerosis (MS) Society and the Spinal Injuries Association. The meeting was observed by a postgraduate researcher who was beginning to work on the JLA pre-term birth PSP. There were attendees from Scotland, Wales, Northern Ireland and England.

Inclusions and exclusions in the process of forming a partnership and appointing a Steering Group frame the accountability of the whole PSP process. In the absence of a constituency as such from which to select and elect a representative steering group, WRPB and JLA approached attendees of the meeting employing the logic of diversity sampling [36]. As the potential decision making body for the PSP it was important that the Steering Group contained the widest range of perspectives to ensure fair deliberation and to enable the partnership to reach as wide a cross section of opinion as possible. The launch meeting agreed that collaboration between parties would be fostered by an independent facilitator from the JLA with no stake in the content of the outcome. The first Steering Group meeting appointed Sally Crowe as its Chair on this basis. Representatives from industry were welcomed but were not eligible to sit on the Steering Group. Once JLAPUP was established it was approached throughout its life by different wound care device companies with tempting offers of resources and other forms of support, none of which were taken up, lest it compromise its independence.

Some members of the Steering Group brought their own direct personal experience of receiving and giving pressure ulcer treatment and care to the table while others attended as representatives of organisations whose members were affected. Two staff from the WRPB and members of third sector organisations who did not have personal or current clinical practice experience of pressure ulcers contributed to discussions but considered it right to abstain from voting on priorities. The Steering Group worked to increase membership of the partnership and developed a process of active and supporting membership. After its initial formation, some members of the group took the option of attending remotely via Skype. This facility was particularly important for those for whom travelling brought the risk of exacerbating pressure ulcer damage.

### Consultation: uncertainties about uncertainty gathering

JLAPUP consultation methods involved giving brief, impartial and sufficiently clear explanations of the process to people often unfamiliar with clinical research and with the concept of uncertainty as the starting point for research. Unless there is uncertainty about the effect of an intervention for pressure ulcer treatment and prevention it is not necessary and therefore not ethical to carry out research. The use of the term ‘uncertainty’ as a noun by the JLA is clear to those familiar with the thinking behind it. However, for those unfamiliar, like Donald Rumsfeld’s famous “known unknowns”, the use of the term can cause confusion. PSPs have a discomforting role in that raising understanding of the concept of uncertainty can serve to undermine people’s confidence in interventions when they realise that they are poorly evidenced.

JLAPUP tried to frame its consultation positively, asking people what it is we need to know in order to improve prevention and treatment of pressure ulcers and where people wished they had answers: ‘if only we knew…’. Articles about JLAPUP and calls for ‘uncertainties’ were placed in a range of media targeted at patients and professionals involved in pressure ulcer care referring back to its website, Twitter account, phone number and email address. Counting each meeting, event (e.g. an interactive stall and canvassing delegates at an MS Society conference), email, tweet, survey response or webinar as one submission source, JLAPUP received 359 submissions from the uncertainty gathering process which together generated a total of 960 uncertainties.

A paper survey was distributed via active members. In order to maximise the chances of reaching patients or service users and carers, and given the absence of specific user groups to target, this was also distributed at NHS sites in the North of England for which we obtained ethical approval. A statement from the National Research Ethics Service (NRES) and INVOLVE states that ethical approval is not needed for the active involvement element of a research project, including identifying and prioritising research topics, even when people are recruited via the NHS [37]. However, this guidance was relatively new at the beginning of JLAPUP and it was decided to apply for approval to ensure we were ‘covered’ and as a means of rigorously thinking through ethical issues including working with potentially vulnerable elders. Ethics approval involved a lengthy and bureaucratic process of engagement with an NHS ethical framework designed predominantly for RCTs [38]. One site granted research approval after the survey closing date prohibiting its distribution there. The number of paper survey returns (83) was disappointing given the investment of time and resources. 49 % were from patients/service users, 16 % from carers and 35 % from health professionals. Against a backdrop of UK austerity cuts and NHS reorganisation, the method relied on already overstretched staff to explain, distribute and collect the survey.

An online survey, accessible nationally, was live for four months. Participants completed the survey as someone having or at risk of pressure ulcers, a carer or a health professional. The aim was to identify any key differences between group submissions for later deliberation. Those who belonged to more than one category were invited to complete the survey from each perspective. Additional limited population data were collected from all respondents. There were 180 online survey submissions (from 344 website visits). Respondents were health professionals (43 %), patients/service users (37 %), carers (10.5 %) and the remainder were from mixed groups. Findings from the WRPB prevalence study indicated that patient/service user respondents to the overall survey were generally younger and more likely to be living at home than the pressure ulcer population as a whole [30]. People in more formal care settings (including people in nursing homes and hospital in-patients) and the UK non-white population were under-represented.

The JLA’s focus is on RCTs and the partnership attempted to keep the focus on uncertainties about the effects of interventions throughout the process. However, many other related concerns and uncertainties about pressure ulcer services and practices were raised. Respondents shared their experiences and problems in narrative form. Many submissions across groups presumed that solutions were already known but not being implemented. Rather than raising uncertainties, these submissions, focused on telling us what should be happening or what had gone wrong. In discussions we were able to listen to the experience, provide explanations about our aims and ask questions which helped to check mutual understanding. Below is an early email and telephone exchange with a carer in response to an article placed in the magazine of the Multiple Sclerosis Society. The initial submission was discussed with the WRPB team and the Chair of the Steering Group to check how the developing JLAPUP approach to interpretation matched approaches in previous PSPs. The replies were made by the WRPB lead who is an experienced qualitative researcher.

Dear Mary

My husband has MS he's had two pressure ulcers for over eight years. One pleased to say is almost healed [In] 2003 when he first got it was so big I could have put my fist in side. The other one which I used to call the small one is between his buttocks it continually bleeds. The District Nurse has tried so hard to get it to heal but it never does? [Name] our District Nurse has used so many different dressings we have used [name of dressing] for years and just ordered [name of dressing] which we have not used before.

My husband [Name] is completely paralyzed with MS can only sit in his wheelchair or lay in bed he has a pressure mattress and two different pressure cushions. He is spending more and more time in bed all due to the pressure sore hurting so much. While in bed it looks as if it’s healing but as soon as he sits it bleeds again.

He goes into [Name of Hospice] for respite so I can have a break which he did two weeks ago on his return home the sore had deteriorated that always happens sadly when I’m not doing the care.

The sore gives him so much pain we are not going out anymore he would rather be in bed . . . Attached photo . . . hope you don’t mind . . .

Dear [Name]

Thank you very much for sharing your experience of caring for your husband and his pressure ulcers.

We are trying to find out which questions about pressure ulcer treatment and prevention carers, patients and clinicians would most like to have answers to. I think your email potentially raises the following questions for MS patients:

How effective is [name of dressing] for the treatment of pressure ulcers?

How effective is [name of dressing] for the treatment of pressure ulcers?

How does the location of a pressure ulcer impact on healing?

Is posture important in relation to healing?

Does the equipment (different types of seats and/or mattresses) work for prevention and/or healing?

Would better pain management increase his desire to get out and about?

Do you think these are the questions you would most like to have answers to? And if so, are there any you think are particularly important? If only we knew..........

You also raise the important safeguarding concern that respite care has led to a deterioration of the pressure ulcer. I will make sure that this is recorded in our findings . . .

With thanks and kind regards

Mary

Contemporaneous notes of follow up telephone call: [Name] interested in questions [above] but would most like to know how to make it heal … feels like, “everyone comes in and looks at it and then goes home. I don’t think anyone has an answer” [also wants to know] if they could do something surgically [which was safe] …

Dear Mary

Thank you for your phone call this morning. I’m so pleased that you are doing research into pressure sores … Doctors tend to put everything down to MS never look at the full picture that it could be something else going on …

Above gives some indication of how people submitted experiences rather than ‘uncertainties’ to the process and the journey in translating the stories they shared into questions for research. This is the challenge of interpreting responses to open-ended questions identified in previous research prioritisation processes [40]. Such initial interactions raised questions about how well people can understand and engage with a remote survey asking for treatment uncertainties or research questions potentially appropriate for RCTs. Another JLA PSP that followed formal protocols that defined a researchable question for RCTs found that it rejected a disproportionate amount of questions submitted by patients and carers, including many understandably hoping for a cure [39]. Note that what the respondent above most wanted to know was how to make pressure ulcers heal.

In the light of the above, the online survey included an optional ‘question builder’ which sought to inform and encourage participants to use the PICO (population/patient, intervention, comparison, outcome) format used in RCT design where possible. 33 % of respondents used the question builder, with 10 % using it for more than one question. There were still many narrative submissions from all stakeholder groups and the survey comments sections were well used. The range of responses included: views on the importance of the uncertainties submitted; comments on the JLAPUP process; requests for information; and further sharing of experience. Requests for advice were submitted throughout the process, although we tried to make it clear on publicity material that it was not appropriate for us to give individual advice.

JLAPUP also attempted to gather uncertainties via a self addressed tear off postcard that was included in a pressure ulcer information leaflet distributed by the Tissue Viability Service at a community healthcare trust. However, instead of identifying areas where they thought there should be more research as requested, most respondents sought medical information or gave an account of how they were managing their risk.

### Collation: further challenges in using methods of interpretation and categorisation

Submissions from all sources were: counted; categorised as intervention or non-intervention questions; re-worked into a PICO format where possible and applicable (noting the journey from initial submission to question); and categorised according to an intervention taxonomy developed from that used by the Cochrane Wounds Group. Duplicates were combined and 960 ‘indicative uncertainties’ were produced.

Submissions were categorised as follows:

- Intervention/non-intervention
- Originator (source)
- Submission group (patient/service user, carer, health professional, mixed)
- Broad intervention taxonomy derived from the Cochrane Wounds group: organisation of care; pressure and shear reduction and relief; local wound treatment; managing patients with limited mobility; risk assessment; nutrition; local skin care; surgery; other.
- Detailed intervention taxonomy derived from the Cochrane Wounds group e.g. local wound treatment (dressings, topical treatments etc.)
- Outcome

The majority of uncertainties related to the effectiveness of interventions (72 %). Of these, the most frequently submitted uncertainties were about methods of pressure and shear reduction and relief (30 %) and the organisation of care (30 %). Prevention was identified as an outcome in 61 % of the intervention uncertainties and healing in 32 %.

Uncertainties that were not about treatment interventions were categorised using the health research classification system of the UK Clinical Research Collaboration [41]. After combining duplicates and removing those too poorly specified to categorise, a list of 220 unique non-intervention questions were recorded. The majority of these were questions about the aetiology or prognosis of pressure ulcers. These were made available on the JLAPUP website with a request for feedback on their use. No such feedback has yet been received.

Checks on the interpretation and categorisation of submissions were conducted by comparing the handling of the same submission by different coders (members of the programme research team and Steering Group members). These were checks for categorisation errors and whether the questions produced were faithful to the submissions without ‘reading-into’ or over-interpreting them. An ‘uncertainty analysis’ was conducted at a Steering Group meeting to scrutinise interpretation of submissions and their translation into PICO style uncertainties. Members of the Steering Group individually checked for ‘justifiability’ of interpretation in a sequential sample showing the journey from submission to question and discussed any disagreements found.

‘Indicative uncertainties’ were then checked against existing systematic reviews to ensure that there was not an existing, reliable answer in the research literature and ensure that identified intervention questions were ‘genuine uncertainties’. An experienced systematic reviewer in this field performed an individualised search for each uncertainty on the Cochrane Database of Systematic Reviews, the Database of Abstracts of Reviews of Effects (DARE) & Technology Assessments Database. Where questions were asked about cost-effectiveness, searches were made for trials and economic evaluations on NHSEED (NHS Economic Evaluation Database). The WRPB PI who was also the Coordinating Editor of the Cochrane Wounds Group, checked the searches. The process and findings were discussed in the Steering Group.

A systematic review found reliable evidence for one uncertainty so this was removed from the list. Partial evidence was found to answer two uncertainties submitted. The limited nature of the evidence meant that these remained on the list of uncertainties published on UK DUETs. Aware of the limitation of its own lifespan, the Steering Group raised the need to update this list over time as the body of evidence develops. They also decided that as the focus of the PSP was collecting and prioritising uncertainties from patients/service users, carers and health professionals, research recommendations from systematic reviews would not be included in the gathering, analysis or prioritisation process because these would be researcher-derived uncertainties.

### Prioritisation: the challenge of producing a workable shortlist and a user friendly survey

The next stage was to reduce the long list of genuine (rather than ‘indicative’) uncertainties that remained after the evidence check to make a workable shortlist for prioritisation. Within separate patient/service user, carer and health professional data sets, questions were ranked and grouped in order of number of times submitted. Where there were strong similarities, some questions were combined. Full criteria for inclusion have been reported elsewhere [3]. There was a tension in this process between producing broader issues for prioritisation and preserving the particularity and authenticity of un-combined questions. The concern about ‘reading into’ submissions was now displaced by a concern about losing specifics. The approach taken tried to balance identifying themes with preserving finer details (populations and interventions). This was an attempt to maintain some of the richness of submissions and the fine detail that would be useful in the design of any future RCTs.

A prioritisation survey was designed in paper and electronic formats to enable stakeholder groups to value the different uncertainties identified during the previous phases by their peers and rank these in order of importance. The aim was to produce group-specific research agendas. At that time, the suggested JLA method depended on people downloading a form, completing it and posting it back rather than submitting online. JLAPUP wanted to ensure that people could submit online because online methods had worked better than paper methods in engaging people so far. Consultations with survey methodologists within the WRPB team included discussions about discrete choice experiments and best-worst scaling techniques. However, writing our own programme was beyond JLAPUP’s resources. We looked at other possibilities within the available software. Some options were unsuitable (pairwise comparisons, for example, needed large numbers of participants). Rating on a Likert scale offered a possible solution that balanced our needs with affordable methods.

The survey design asked people to rate uncertainties, expressed as research questions, according to their importance on a scale of 1 to 10 to encourage greater differentiation in responses, with an option for “No view” or “Don’t know”. The question order was randomised. Limited population data were collected, in line with the gathering survey, but with an added question about wheelchair use. The survey was amended after feedback from a panel of patients/service users on ease of use and clarity of understanding and measures were taken to safeguard security and prevent multiple entries. Online and paper surveys were open for five weeks and were launched with a widely distributed press release and a social media campaign.

Explanatory text for the online survey was revised a number of times in the light of comments received from respondents. Some had difficulty prioritising the uncertainties, “[y]ou say not to rate all the questions as ‘very important’ but that is difficult when they are!” This comment from a patient/service user was echoed in others from health professionals and carers. Some wanted to answer the questions rather than choose which should be researched and the concept of uncertainty remained difficult to explain. On the basis that people were not necessarily reading, or if they were, understanding the explanatory text, a brief explanatory YouTube video was created and linked to the online survey and JLAPUP website.^2^ This was viewed 90 times and had an impact in reducing the number of calls and email queries.

Paper survey responses were input into the online survey and three data sets were produced using Stata for patient/service user, carer and health professional respondents. 141 participants completed the surveys rating the majority of uncertainties as of above average importance. 50 % of respondents were health professionals, 40 % patients/service users and 10 % carers. 80 % of patient/service user respondents said that they were wheelchair users, 93 % were living at home independently or supported by family or carers. 59 % were aged 40–64. Of the carers, 75 % were caring for someone who uses a wheelchair; 75 % for someone living at home, and 44 % for someone aged 40–64.

### Challenges posed by integration and deliberation

The first stage of the integration phase involved identifying the similarities and differences in responses from the separate stakeholder group prioritisation. Questions that covered similar areas but were asked by different groups were thematically combined. As broader questions emerged, there was an attempt to retain some of the granularity, the small differences and shades of meaning that was in danger of being lost through combination. The production of one overarching topic/question for the priority setting process potentially loses the richness of what partners are trying to say and the specific detail useful in planning research. Table 1 shows some of the detail behind the finally selected top JLAPUP priority, *How effective is repositioning in the prevention of pressure ulcers?*

**Table 1:** Detail behind the JLAPUP top priority

How effective is repositioning in the prevention of pressure ulcers?		
Population	People of all ages at risk of pressure ulcers in bed.	People of all ages at risk of developing a pressure ulcer who have limited mobility and are largely seated.
Sub groups	Those with contracted limbs, dementia, heel pressure ulcers, incontinence, stroke, spinal injury, multiple sclerosis, bariatric, cachexic, in hospitals and institutions including surgical patients and those nursed in bed and those cared for at home.	Wheelchair users, with for example co-morbidities such as multiple sclerosis and spinal cord injury.
Interventions and comparisons	The optimum frequency for turning e.g., two hourly turning vs. four hourly turning in relation to the type of mattress or surface being used.	The optimum frequency and techniques for changing position or posture by self or others.
	The relative effectiveness of methods of repositioning broader than turning e.g., the Trendelenburg system of positioning vs. the knee break system or the effectiveness of 30 degree tilt in conjunction with pressure relieving mattress.	Comparisons of techniques and tools for limiting damage when transferring between surfaces and lifting off cushions.
Outcomes	Prevention of pressure ulceration.	Prevention of pressure ulceration.
	Impacts on quality of life of patient, carer and partners over 24 hour period, including sleep disruption and the impacts on those who would usually bed share.	Impacts on quality of life of patients and carers including the ability to undertake usual domestic, social and work/education activities. Limiting damage caused when transferring between surfaces.

There were strong shared levels of agreement between stakeholders but also a number of uncertainties had been identified and ranked by one group only. If agreed in this stage as important by all stakeholder groups, selections were included in the final shortlist of uncertainties for deliberation at a final priority setting meeting. In addition, this phase also gave people the opportunity to consider the importance of, and a chance to include, uncertainties that may not have occurred to them from their own patient or service user, carer or health professional perspective. Selections were made in the Steering Group and more widely using two methods. A facilitated voting sheet exercise was conducted at events on Worldwide STOP Pressure Ulcer Day. These events attracted more health professionals than patients/service users or carers. 50 health professionals, six patients/service users and four carers ranked the three most important prevention and three most important treatment uncertainties which had been submitted by one stakeholder group only. Eight members of the new Pressure Ulcer Research Service User Network (PURSUN UK) took part in a workshop to hear about the process so far, identify any gaps and make their selections. Participants selected liberally across originating groups.

The second stage of the integration phase involved home and bedside interviews with care home residents. Although key stakeholders, they are often not included in studies about older people’s health [42] and were under-represented in our consultation phase. Visits to care homes and a rehabilitation centre were arranged to ask which of the treatment/prevention uncertainties gathered so far mattered most to residents and whether they had others they would like to include. Permission was granted to access three sites all of which were proud of their low recordings of pressure ulceration. Two sites were visited; the rehabilitation centre could not identify anyone at risk who was able to participate. Although very willing to speak informally, most residents of the care homes we approached did not want to deal with the paperwork made necessary by the conditions of ethics approval. The largest group of people at risk of pressure ulcers are the frail elderly with reduced mobility. When we did talk to one or two people who were ‘at risk’, it was difficult to engage them in a conversation about something that might happen to them. Their immediate concern was the multiple comorbidities that were already impairing their health. A conversation about something that might happen, but had not yet, and might never happen to them, proved too abstract. The Steering Group agreed that this aspect of the work was important due to the effective disempowerment of people in care settings. They also agreed that JLAPUP did not have the time and resources to meet the complexity of the task and that this was an area that should be addressed in future research.

The final stage of the integration phase was a deliberative priority setting meeting to choose the top ten intervention uncertainties for future research. All active members and supporters and those who had taken part in the process to date who had submitted their details and expressed an interest in future involvement were invited to take part. The format of the meeting was adapted from the existing format for final JLA meetings, based on nominal group technique, with new ideas piloted in mini mock workshops.

Before the meeting, participants were asked to complete a pre-workshop exercise to choose and rank their individual top ten preferred uncertainties from the thirty most frequently submitted and highly ranked questions. The meeting itself was staffed by: facilitators; a statistician to rank uncertainties; a qualitative researcher who hosted a “listening/experience corner” (an opportunity for those who wanted to talk or tell their story outside the confines of the demands of the workshop); and an independent observer. Researcher members of the meeting were non-voting and were asked to act as jargon de-buggers and to offer research clarification where this was requested from members of the meeting. The backstory to all uncertainties under consideration and a Cochrane Wounds Group glossary of key terms in wounds research were provided. The meeting began with a brief explanation of what had happened to submissions and in order to keep the focus on intervention uncertainty, a reminder of why identifying uncertainties was important i.e. that doing things that have not been tested can cause harm even when the intentions are good and that spending money on ineffective interventions means less funding for finding and implementing things that work.

The meeting went well and successfully agreed a top twelve, but there were tensions, including some service users and carers feeling they were not being heard by particular health and research professionals. The deliberative ideal requires: equal citizenship; equality in resources (in order to get to the table in the first place, presuming one is well enough to attend); equal distribution of the skills with which to make persuasive arguments and equality in “epistemological authority” i.e. the “capacity to evoke acknowledgement of one’s arguments” ([43]: 348). This is hard to achieve in any ‘mixed’ UK forum given: the increase in social inequalities which map onto health inequalities [44]; uncertainty about the public role in and understanding of health research [45]; and the history of hierarchy, paternalism and top down view of knowledge production in both medicine and academia. Group discussions were audio recorded. Subjects for further exploration include the dynamics of expertise and the extent to which service user perspectives were subject to conscious or unconscious co-option in public dialogue with professionals [46]; the extent to which dominant individuals may have persuaded through emotivism; whether conventionalism dominated (perpetuating received ideas and reinforcing how things are usually done); the impact of the imbalance in technical knowledge; and how successful the meeting was in communicating in technical, lay and professional languages.

Research into interventions for pressure ulcer prevention was identified as an important gap in the consultation phase. However, in this deliberative phase, research into treatment was also given high priority on the basis that some pressure ulcers are unavoidable and in light of the enormous level of uncertainty about treatment effectiveness in this area. In keeping with previous PSPs, JLAPUP raised questions about the effectiveness and harmonisation of NHS service models and the best means of supporting patient and family self care within those models [47].

### Uncertainties about impact

The top twelve uncertainties identified by JLAPUP were widely publicised. Measuring the final impact of the process is contingent on the methods and outcomes of funders’ and commissioners’ often opaque decision making processes. Representatives from NETSCC attended a Steering Group meeting with a view to developing a commissioning brief. This revealed a disconnect between the Steering Group and NETSCC about whose ‘job’ it was to focus broad uncertainties back down into tightly focused, PICO-based uncertainties.

As noted, the process of combining questions for prioritisation results in broad theme based questions tends to lose the granularity and specificity which makes for a clear, researchable question. NETSCC had been provided with a breakdown of the aspects of the topic raised by participants in its process but was hoping that the Steering Group would take the broad top twelve uncertainties identified and do some further work to focus them down. However, the Steering Group and its support staff were not resourced to continue beyond the funding of the WRPB programme and the Steering Group was not a representative group with a wider mandate to speak on behalf of a pressure ulcer constituency. It did not feel authorised to speak on behalf of people with pressure ulcers in a wider context. It had been appointed to oversee and disseminate the outcome of a now completed prioritisation process and did not think it was its role to further second guess or re-interpret what was intended by those who participated in the uncertainty generation and prioritisation. It felt that taking this further step into translation and focusing threatened to negate the work that had been done to make JLAPUP methods as rigorous, transparent and accountable as possible at each stage. Instead, the Steering Group felt strongly that the research community should be invited to respond to whichever of the priorities funders wanted to take further and that, in so-doing, it was up to research teams to justify their interpretation of the priority. It was recommended that any successful research bid against a JLA priority demonstrate how patients/service users have been involved in interpreting the priority and developing the proposal.

Unsuccessful attempts have since been made to follow up progress from this meeting. Once funding has stopped and the Steering Group no longer meet the PSP is effectively finished. However, the process of publicising the priorities and ensuring they are translated into research is inevitably a longer term game. There is uncertainty about the extent to which concerns and questions posed in the process are being heeded and their suitability for research.

## Conclusion

PSPs provide a snapshot of the most important unanswered questions as identified by a collectivity of those affected by, supporting and representing those with a condition; a strategic public. This consultative, deliberative stakeholder process does not incorporate broader societal considerations for research priority setting. Facilitators of the JLA PSPs are tasked with managing values and perspectives and distinguishing patient/service users’, carers’ and health professionals’ individual priorities based on personal experience from priorities which potentially have an impact on a larger group of people and may reduce the collective burden of a health problem. Funders and commissioners must use other mechanisms to decide among competing priorities in relation to existing priorities, research feasibility, cost and potential public health benefit. The following challenges for PPI identified by Clark et al. [48] are therefore of key relevance to understanding and evaluating the processes and impact of each JLA PSP: the contestation between professional and lay power/knowledge; the reconciliation of needs, wants, rights and resources; the reconciliation of agendas about choice with those of equity; and the implications of the debate about ‘responsibilising’ patients, carers (and clinicians) to make choices (even if undesired).

The JLAPUP process showed that it was possible for stakeholders in the field of wound care to work in partnership. It aimed to track the similarities and differences in views throughout the process, however there proved to be less opportunity for direct dialogue and deliberation than initially hoped because this was the most expensive and time-consuming aspect of the process. Nevertheless, it did open up discussion and began to explore the gap between patient/ service user experience, and health professional and researcher understanding of what is of most importance and also, the extent to which the solution to these concerns rests on identifying the appropriate RCTs. In addition to the effectiveness of treatment and prevention interventions, JLAPUP stakeholders were concerned to see more research on aetiology, diagnosis, prognosis and other aspects of care.

JLAPUP revealed the level of difficulty of structuring meaningful conversations with patients/service users, carers and health professionals about uncertainty as a concept in treatment and research. It also revealed the difficulty for all stakeholders in acknowledging that some strongly held beliefs about wound care are actually big research uncertainties. There is an understandable tendency to want to feel that one is doing the right thing and facing uncertainty in undergoing or delivering treatment and prevention is difficult. In the context of treatment for cancer, Fallowfield et al. [49] identify, “an inability to predict the outcome of events [as] a psychonoxious experience for anyone.”

Decision making in JLAPUP was carried out by patients/service users, carers and health professionals working together in a complex co-production process supported by a PPI officer and researchers with expertise in participatory research methods, systematic reviews, survey design and the conduct of RCTs. Health researchers are excluded from the JLA PSPs on the grounds that they have vested interests in setting the research agenda, yet the process is about research and involves a variety of research skills. If the JLA PSPs are successfully managing to get health professionals to work in partnership with patients and service-users, there may also be hope that they can harness appropriate researcher expertise and resources in support, rather than co-option, of PSPs.

The JLA project of getting patients and clinicians to work in partnership to hold the research community to account is an ambitious and challenging one. The JLA PSPs are complex interventions which make an important contribution to thinking about public participation in research for healthcare and what constitutes good decision making. They emerge within, and to some extent against, a narrow biomedical focus which neglects social and economic determinants and contexts of health and which can lead to a focus on the treatment of body parts at the expense of individual patient care. JLA PSPs originate from the evidence-informed healthcare movement predominantly associated with (post) positivist epistemological assumptions and objective, statistically derived data. They also straddle the worlds of social and medical research. Understanding the processes of health research decision making and holding the research community to account involves: paying attention to qualitative methodologies; to the changing history of the public sector; and the social and political context of health care knowledge production, which, in the UK currently includes moves to privatise socialised medicine and the promotion of products by multinational companies at the expense of public health.

## Footnotes

1 “Clinician” is the term used by the JLA but because this is interpreted by some people narrowly as “doctor”, JLAPUP used the broader term “health professional”.

2 https://www.youtube.com/watch?v=Cw4DhnWwnjc
